# Supplementary material for: A Novel Bispecific Antibody Targeting CD3 and Lewis Y with Potent Therapeutic Efficacy against Gastric Cancer
Source: Biomedicines. 2021 Aug 20;9(8):1059. doi: 10.3390/biomedicines9081059 (PMC8393954; doi:10.3390/biomedicines9081059)
Supplement: Supplementary file 1 [file biomedicines-09-01059-s001.zip › biomedicines-1257833-supplementary.pdf]

Supplementary materials

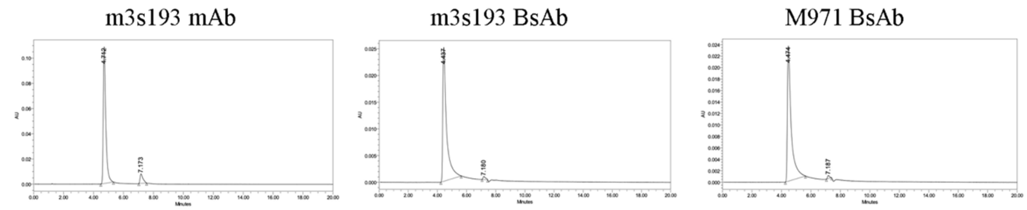

Figur S1. SEC-HPLC analysis of m3s193 mAb, m3s193 BsAb and M971 BsAb. Samples were flowed by 6 × 40mm Guard Column (Ultrahydrogel™) and 7.8 × 300mm Column (Ultrahydrogel™ Linear), and using Gel Permeation Chromatography (GPC) to analyze. Major peak (about 4 minutes) is the fully paired mAb (molecular weight 150 kDa) and BsAb (molecular weight 200 kDa), and salt buffer peak (about 7 minutes).

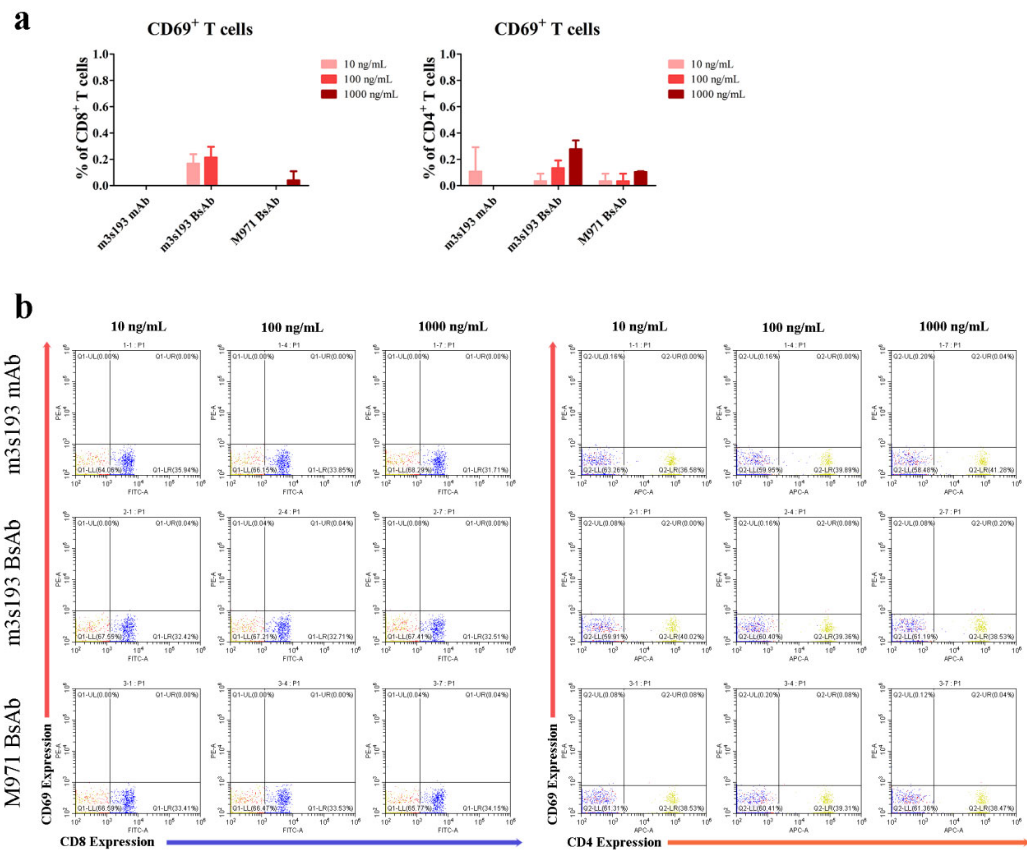

Figure S2. Auto-activation of huPBMCs mediated by m3s193 BsAb. HuPBMCs were co-incubated with mAb or BsAb under the concentrations of 10, 100, 1,000 ng/mL. After 20 h incubation, percentages of $CD8^+CD69^+$ on $CD8^+$ T cells and $CD4^+CD69^+$ on $CD4^+$ T cells were calculated by flow cytometry through FITC-CD8, APC-CD4 and PE-CD69 mAbs, and analyzed by GraphPad Prism. (**a**) Histogram of percentage of CD69 positive T cells on $CD8^+$ or $CD4^+$ T cells. (**b**) Flow cytometry assay of CD69 expression on $CD8^+$ and $CD4^+$ T cells.
